# Preparation and Characterization of Electrospun PAN/PSA Carbonized Nanofibers: Experiment and Simulation Study

**DOI:** 10.3390/nano8100821

**Published:** 2018-10-11

**Authors:** Shixin Jin, Jiali Yu, Yuansheng Zheng, Wen-Yi Wang, Binjie Xin, Chi-Wai Kan

**Affiliations:** 1 School of Fashion Engineering, Shanghai University of Engineering Science, Shanghai 201620, China; jinshixinyishui@163.com (S.J.); yuansheng.zheng@outlook.com (Y.Z.); 2 Institute of Textiles and Clothing, The Hong Kong Polytechnic University, Hung Hom, Kowloon, Hong Kong, China; m15021818332@163.com (J.Y.); tcwang@polyu.edu.hk (W.-Y.W.)

**Keywords:** electric field simulation, electrospinning, carbonized nanofibers, conductivity

## Abstract

In this study, we simulated the electric field distribution of side-by-side electrospinning by using the finite element method (FEM), and studied the effects of spinneret wall thickness, spinning voltage and receiving distance on the distribution of the electrostatic field. The receiving distance was selected as a variable in the experimental, a series of PAN/PSA composite nanofiber membranes were prepared by using a self-made side by side electrospinning device. The membranes were tested by Fourier-transform infrared (FTIR), thermogravimetric analysis (TG), and scanning electron microscope (SEM). The prepared membranes were also treated by high-temperature treatment, and the change of fiber diameter and conductivity of the membrane before and after high-temperature treatment were studied. It was found that the PAN/PSA carbonized nanofibers could achieve a better performance in heat resistance and conductivity at 200 mm receiving distance.

## 1. Introduction

Due to its unique versatility, composite nanomaterials have attracted great attention in many fields—such as solar cells, sensors, magnetism, luminescence, catalysis, medical treatment, and other numerous fields [1,2,3,4,5,6]. Electrospinning has been recognized as one of the most commonly used methods for the fabrication of composite nanomaterials and the mass-preparation of nanofiber materials [7,8,9]. Many factors (such as solvent, spinning voltage, flow rate of spinning solution, receiving distance, spinning temperature, and humidity, etc.) affecting the morphology of electrospun nanofibers have been studied by a lot of groups in recent years, respectively [10,11,12,13,14,15,16,17]. In addition, side by side electrospinning, as a type of electrospinning is widely used to prepare nanomaterials, and many studies are based on this method [8,18,19,20].

Zeng et al. [21] presented a novel dual-spinneret which can be used to prepare Janus-type nanofiber with thermoelectric p–n heterostructure, and this Janus-type structure could be widely used as a p–n junction in nanoscale thermoelectric devices. Yu et al. [22] employed a Teflon-coated side-by-side spinneret to fabricate highly tunable biphasic drug release medicated Janus fibers. The coated spinneret guaranteed the formation of a Janus Taylor cone and high-quality integrated Janus structures. Zhu’s research group [23] provided five kinds of dual-spinnerets to study the effect of side-by-side spinneret on the fabrication of composite nanofibers via electrospinning. It is found that the structure of side-by-side spinneret had an obvious effect on the preparation of p-CuO/n-TiO_2_ composite nanofibers Chen et al. [24] found that the structures of the Janus nanofibers could be manipulated easily by varying the port angle and they prepared a series of structure-tunable Janus nanofibers via side-by-side electrospinning varying port angles spinnerets. Peng et al. [25] prepared two-in-one composite fibers with side by side arrangement of silk fibroin and poly(l-lactide) by electrospinning. However, there is insufficient research conducted on the influences of electric field on the side by side electrospinning.

Polysulfone amide (PSA) is a representative polymer for new-type high-temperature resistant fibers, and the micron-sized PSA fibers have been widely used [26,27,28,29]. Polyacrylonitrile (PAN) is a conventional chemical fiber and is an important material for the preparation of carbon fiber precursors [30,31,32]. Carbon nanofibers are made by carbonization of their precursors of electrospun nanofibers with many excellent properties such as superior mechanical strength, adsorption performance, electrochemical performance, and so on [33,34,35,36,37,38]. Although many studies have been conducted on the preparation of PAN and PSA nanomaterials respectively [39,40,41,42], there are quite a few studies that focus on the PAN/PSA composite materials and the PAN/PSA composite carbonized materials.

In the present study, the effect of electric field distribution on the yarn formation and the properties of electrospun PSA nanoyarns were systematically studied [20,29]. Some factors—including spinneret size, spinning voltage, and receiving distance—that affect the side by side electric field distribution were studied and the nanomaterials prepared by changing the receiving distance were characterized in this manuscript.

## 2. Experiment and Simulation

### 2.1. Experimental Materials

**Figure 1:**
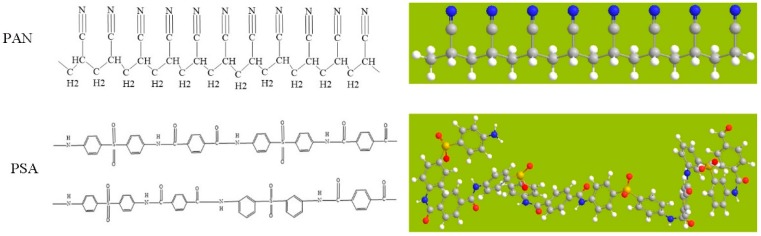
Molecular formulas and 3-D molecular structures of the PAN and PSA.

Polyacrylonitrile (PAN), with an average molecular weight (Mw) of 90,000, was obtained from Sigma-Aldrich Co., Ltd., Saint Louis, MO, USA. Polysulfonamidefiber (PSA), with an average molecular weight (Mw) of 150,000~200,000, was obtained from Shanghai Tanlon Fiber Co., Ltd., Shanghai, China. Figure 1 shows the molecular formulas and 3D molecular structures involved in this work. N, N, -Dimethylformamide (DMF) and Dimethylacetamide (DMAc) were purchased from Sinopharm Chemical Reagents Co., Ltd., Shanghai, China. All chemicals were used as received without further purification.

### 2.2. Fabrication of PAN/PSA Carbonized Nanofibers

The precursor solutions of PAN and PSA with the concentrations of 12 wt% were prepared by dissolving the PAN and PSA in corresponding solvents respectively. PAN was dissolved in DMF, meanwhile PSA was dissolved in DMAc. PAN and PSA with the weight ratios 1:1 are prepared to form the spinning solution. All experiments were performed at about 25 °C and 40~60%RH.

**Figure 2:**
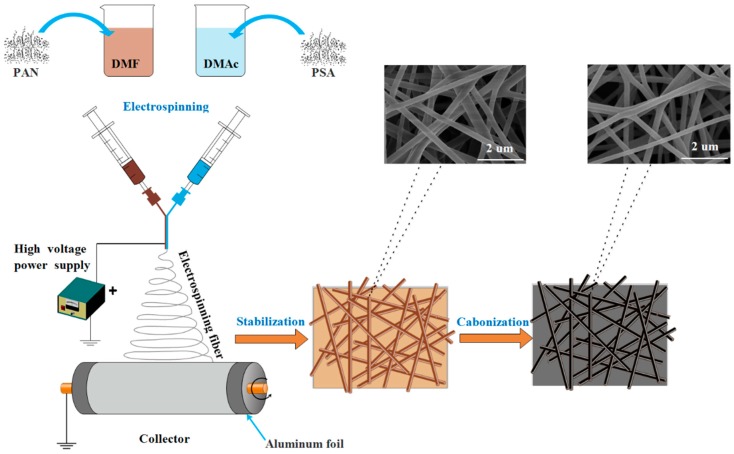
Schematic illustration of the system and the forming of carbonized nanofibers.

The PAN/PSA carbon nanofibers were prepared through side by side electrospinning and subsequent carbonization. A side by side spinneret was used for electrospinning as illustrated in Figure 2. The spinneret consists of two symmetrical needles, to generate nanofiber membranes with a consistent thickness, the spinneret can be moved reciprocally in the horizontal direction, the alternating speed was kept at 200 cm/min, and a rotating drum was set as collector. The receiving distances were set as 100, 150, and 200 mm respectively. The spinneret was stainless steel with inner diameter of 0.5 mm and outer diameters of 0.8 mm with a needle thickness of 0.15 mm. The spinning voltage was 20 kV, the flow rate was 0.15 mL/h and the rotating speed of the collector was 100 rpm.

The obtained precursor PAN/PSA nanofiber membranes were first treated at 80 °C for 1 h to remove the solvent and then stabilized at 280 °C in air for 2 h. Afterwards, the obtained nanofibrous membranes were calcined at 800 °C for 2 h with the heating rate of 5 °C/min under N_2_ flow in the tube furnace, and the carbonized membranes denoted was PAN/PSA CNFs.

### 2.3. Characterization

#### 2.3.1. Scanning Electron Microscopy

The morphologies of the PAN/PSA CNFs were observed by scanning electron microscopy (SEM, SU8010, Hitachi, Tokyo, Japan). In order to form a high-quality image, the sample is sputter coated with a thin layer of gold (~5 nm), the magnification was 20 K. The diameters of the fibers were calculated based on SEM images using image-analysis software (Image Pro Plus 6.0, NIH Image, Bethesda, MD, USA).

#### 2.3.2. Fourier-Transform Infrared Spectroscopy

The chemical structures of the membranes were analyzed by using Fourier-transform infrared spectroscopy (FTIR, PerkinElmer, Waltham, MA, USA) with KBr crystal in the infrared region 5000–400 cm^−1^ at a resolution of 4 cm^−1^. The stretching vibration between 2260 and 2210 cm^−1^ is the best indicator for identifying nitrile groups, the stretching vibration between 3500 and 3300 cm^−1^ is the best indicator for identifying amine groups and the stretching vibration between 1170 and 1140 cm^−1^ is the best indicator for identifying sulfone groups [43,44,45].

#### 2.3.3. Thermal Analysis

The thermal stability of the membranes was carried out using both differential scanning calorimetry (DSC, PerkinElmer, Waltham, MA, USA) and thermogravimetric analysis (TGA, PerkinElmer, Waltham, MA, USA). DSC curves of the membranes were obtained using a DSC 4000 by heating from −60 °C to 350 °C in N_2_ at a heating rate of 5 °C/min with nitrogen flow rate at 20 mL/min. TGA temperature range was from 30 °C to 850 °C at a heating rate of 5 °C /min with a sample about 5 mg, and the nitrogen flow rate was 20 mL/min.

#### 2.3.4. Electrical Conductivity

Electrically conductivity of the PAN/PSA CNFs was measured by using a four-point probe system with a linear probe head (SZT-2C, Soochow, China), each membrane was measured five times in the vertical and horizontal direction, and the average value was used for evaluation, the tests were carried out in a constant temperature and humidity chamber with a temperature of 25 °C and a humidity of 50 °C.

### 2.4. Electric Field Simulation

**Figure 3:**
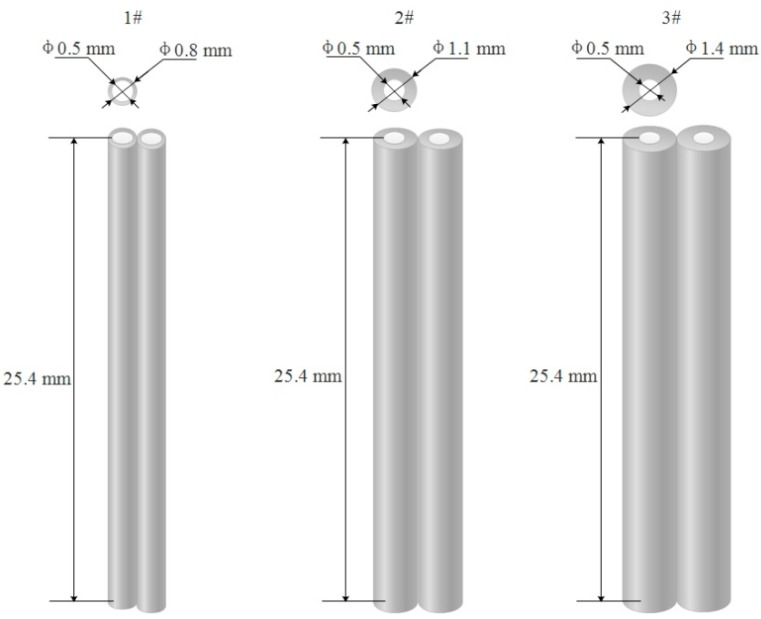
Schematics of spinnerets simulation in the electric field with different needle thickness.

The three-dimensional (3D) electric fields generated from the spinneret to collector were analyzed by COMSOL Multiphysics (version 5.2 a, COMSOL Inc., Stockholm, Sweden) software using the finite element method (FEM). The physical geometries of the electrospinning setups (e.g., spinnerets, receiving distance and collector) and polymer solution were established according to their practical dimensions, locations, and relative permittivities. The electric field simulation with different spinnerets needle thicknesses (0.15, 0.3, and 0.45 mm), different receiving distances (100, 150, and 200 mm), and different spinning voltages (20, 25, and 30 kV) were simulated respectively. The different spinnerets and the simulations of spinneret needles with different thickness were as shown in Figure 3. When performing electric field simulation calculation, the free tetrahedral mesh was used to split the mesh in a finer size and the calculation was performed under steady state conditions.

## 3. Results and Discussion

### 3.1. Simulation Results and Analysis

#### 3.1.1. Effect of Needle Size on Electric Field

**Figure 4:**
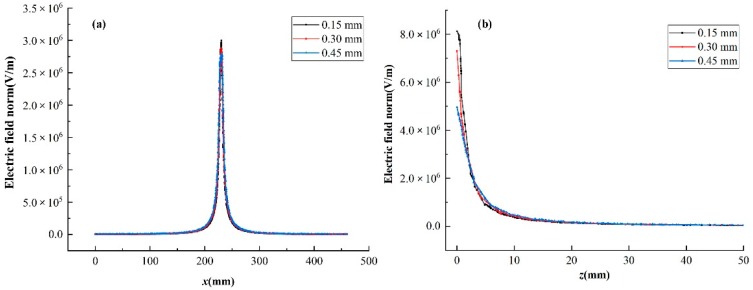
Electric field intensity of electrospinning setups for different needle thickness with a receiving distance of 150 mm. (**a**) electric field intensity along the *x*-axis at 2 mm below the needles and (**b**) electric field intensity along the *z*-axis.

The distribution of electric field is an important factor that affects the electrospinning process. For comparison, the electric fields effected by the three types of needle size were simulated. Figure 4 shows the electric field intensity along the *x*-axis at 2 mm below the spinneret and along the *z*-axis from the spinneret to the collector with three different needle thickness. As illustrated in the Figure 4a, along the *x*-axis, the electric field intensity increased firstly to the maximum at the spinneret position and then decreased symmetrically, with the increase of the thickness of the spinnerets, the electric field intensity strengthened except at the position of the spinneret, and the most obviously change was at the top of the spinneret, and the intensity decreased from 2.96 × 10^6^ V/m to 2.8 × 10^6^ V/m with 5.4 presents reduction. The increase of needle thickness expands the volume of the electric field generator which in turn increases the electric field intensity while it makes the electric field distribution more uniform with a small attenuation of the electric field intensity at the spinneret along the *x*-axis. The change of the electric field along the *y*-axis is the same as the *x*-axis. The electric field intensity along the *z*-axis was also affected by the change of needle thickness, it was strengthened with the needle thickness increase except at the spinneret and the collector. Figure 4b shows the electric field intensity along the *z*-axis from the spinneret to the collector, corresponding to the electric field intensity along the *x*-axis. At the spinneret, a strong electric field concentrates on this surrounding area, when the needle thickness is small, the electric field intensity is large. Finally, the electric field intensity decreased almost to zero at the edge of the collector. The increase of the needle thickness increases the electric field intensity and improve the electric field uniformity, which is more convenient to form a stable and uniform electric field in the electrospinning process.

#### 3.1.2. Effect of Spinning Voltage on Electric Field

**Figure 5:**
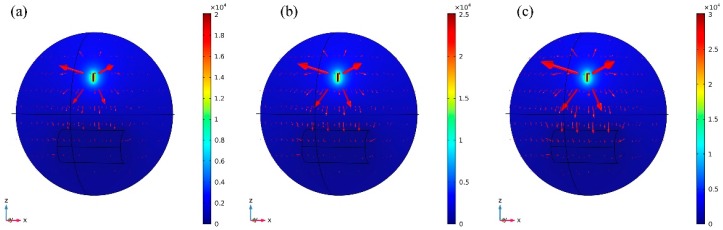
Electric field distribution at different voltages with receiving distance of 150 mm: (**a**) 20 kV, (**b**) 25 kV, and (**c**) 30 kV.

Spinning voltage is also a very important factor affecting the electric field of electrospinning. Figure 5 presents the 3D electric field and potential distributions between the spinneret and the collector with a spinneret specification of 1# and the receiving distance of 150 mm, three different spinning voltages (20, 25, and 30 kV) were applied. The slice plots show the electric potential distribution, and the arrows indicate the field distribution. As can be seen in the Figure 5, the electric field direction is from the spinneret point to the collector, and at the area of spinneret, there was a higher electric field intensity meanwhile the electric field intensity at the collector is rather weak.

The static electric field was calculated using Equation (1), where *ε* is the permittivity, *ρ* is the space charge density and the ∇*V* is the spinning voltage.

(1) −∇·(ε∇V)=ρ

**Figure 6:**
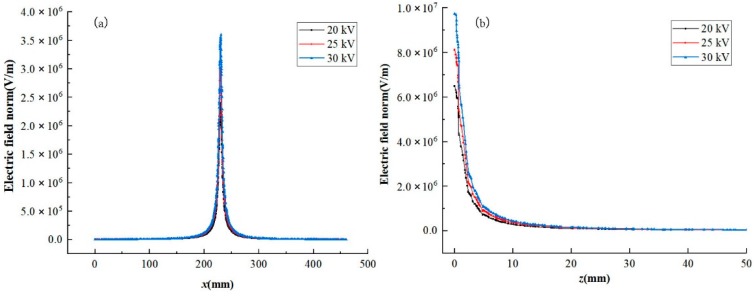
Electric field intensity of electrospinning setups for different voltages with a receiving distance of 150 mm; (**a**) electric field intensity along the *x*-axis at 2 mm below the needles and (**b**) electric field intensity along the *z*-axis.

As shown in Equation (1), the *ε* and *ρ* are constant, the electric field intensity is only determined by the ∇*V*. The electric field intensity under different voltages are as shown in Figure 6, when the spinning voltage increases, the electric field intensity changes significantly along both the *x*-axis and *z*-axis, especially at the area around the spinneret. A higher spinning voltage makes the electric field intensity stronger, which leads to more energy being obtained for the spinning process.

#### 3.1.3. Effect of Receiving Distance on Electric Field

**Figure 7:**
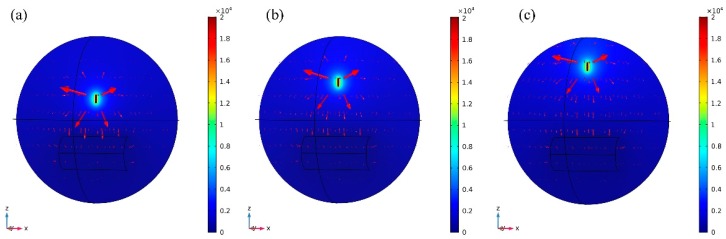
Electric field distribution at different receiving distances with voltage of 20 kV: (**a**)100 mm, (**b**) 150 mm, and (**c**) 200 mm.

In the electrospinning electric field, receiving distance also affect the distribution of the electric field. When the receiving distance is short, the polymer jets movement is unstable, some beads appeared on the membranes, while long receiving distance is suited to prepare nanofibers with smaller diameters. In this study, three receiving distances between the spinneret and the collector were studied, and the spinning voltage was set as 20 kV and the receiving distances were 100, 150, and 200 mm respectively. Figure 7 shows the 3D electric field and potential distributions between the spinneret and the collector with a spinneret specification of 1#. It can be seen that the electric field direction is from the needles point to the collector and concentrated at the edge of the collector. Although the total field distribution trend appears identical all three cases, the local electric field distribution is significantly different. The subsequent experiments were also performed with these three receiving distances.

**Figure 8:**
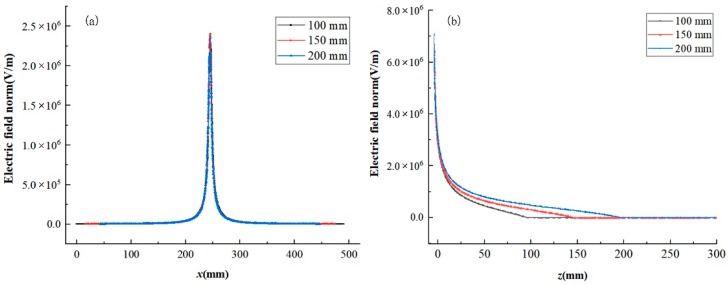
Electric field intensity of electrospinning setups for different receiving distances with a spinning voltage of 20 kV; (**a**) electric field intensity along the *x*-axis at 2 mm below the needles and (**b**) electric field intensity along the *z*-axis.

As illustrated in the Figure 8a, along the *x*-axis at 2 mm below the needles, the receiving distance has no obviously effect on the electric field intensity. Along the *z*-axis, the same effect on the intensity of electric field affected by three different receiving distance could be obtained, with the intensity decreasing from 7.1 × 10^6^ V/m to 2.0 × 10^6^ V/m at almost 15 mm below the needles. Then with the increase of the receiving distance, the decreasing rate of electric field intensity decreased, which means that when the receiving distance is 200 mm, the electric field still has a relatively high intensity at the position of 100 mm to 200 mm along the *z*-axis, it provides a guarantee for the drafting of the fibers to form finer fibers.

### 3.2. Chemical Structure of Samples

**Figure 9:**
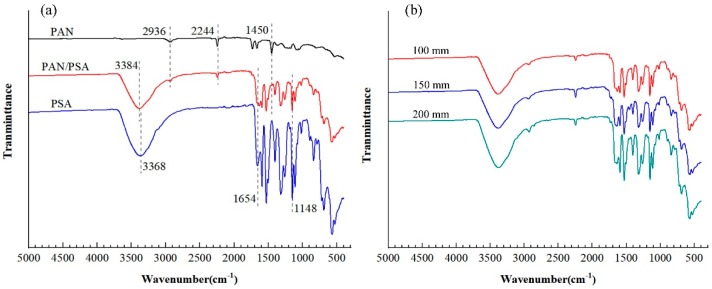
FTIR of different membranes: (**a**) prepared with different materials; (**b**) prepared at different receiving distances.

Fourier-transform infrared measurement is an effective mean to reflect some information about the chemical composition of the fibers. The composition of fiber membrane and the effect of receiving distance was confirmed by FTIR spectra as shown in Figure 9. It can be observed from Figure 9a that, in the spectrum of pure PAN fiber, the peak at 1450 cm^−1^ is the characteristic C–H stretching band of methylene and the peak at 2936 cm^−1^ of C–H stretching vibration. The vibration at 2244 cm^−1^ belongs to C≡N. In the spectrum of pure PSA fiber, the peak at 3368 cm^−1^ is the characteristic N–H stretching band of amide, in addition, the peak at 1148 cm^−1^ is the S=O stretching vibration and the peak at 1654 cm^−1^ is the C=O stretching vibration, the absorption peaks at 1504, 1528, and 1590 cm^−1^ belong to benzene ring skeleton vibration. After combining PAN and PSA by side by side electrospinning, both the main peaks of PAN and PSA can be found in the infrared spectrum of the composite fiber membrane, some slight shifts happened such as N–H stretching band of amide shifted from 3368 cm^−1^ to 3384 cm^−1^.

Figure 9b is infrared spectra of PAN/PSA nanofiber membranes with different receiving distances. As can be seen from the Figure 9b, the number of characteristic peaks and the position of the base did not change a lot, this indirectly shows that the change of receiving distance has no obvious effect on the chemical structure of the membranes.

### 3.3. Surface Morphology

**Figure 10:**
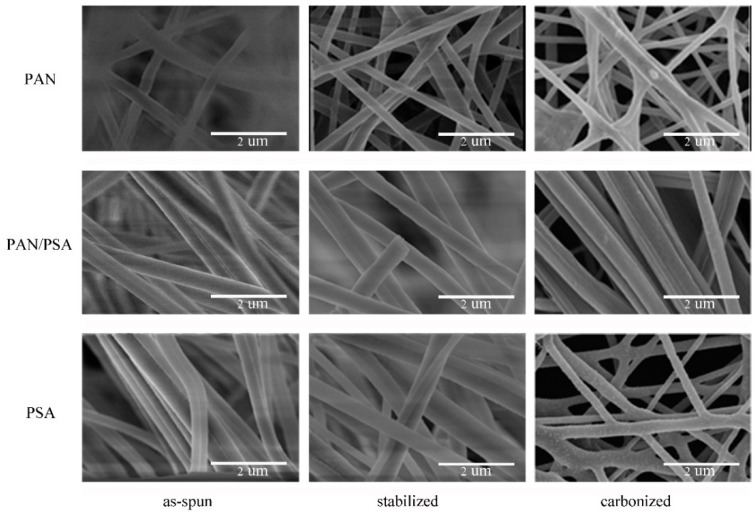
SEM image of membranes of different materials at different processing stages: as-spun, stabilized, carbonized.

After stabilization and carbonization, the morphology of nanofiber membrane was changed. Figure 10 shows the different morphology of membranes before and after carbonization at different processing stages: as-spun, stabilized, and carbonized. The gaps between the fibers in the as-spun membranes were larger and the fibers were fluffier. After stabilization, the fibers are no longer fluffy, the adhesion among the fibers also decreases. There are some fusion and re-adhesion occurred at the fiber intersections after carbonization.

**Figure 11:**
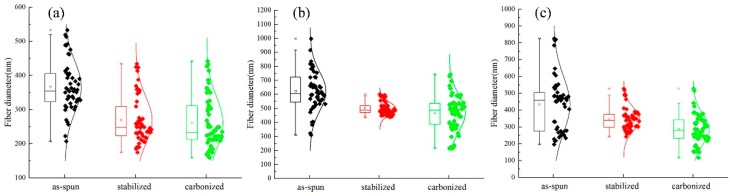
Comparison of the fiber diameter of PSA of different materials at different processing stages: (**a**) PAN; (**b**) PAN/PSA; (**c**) PSA.

**Figure 12:**
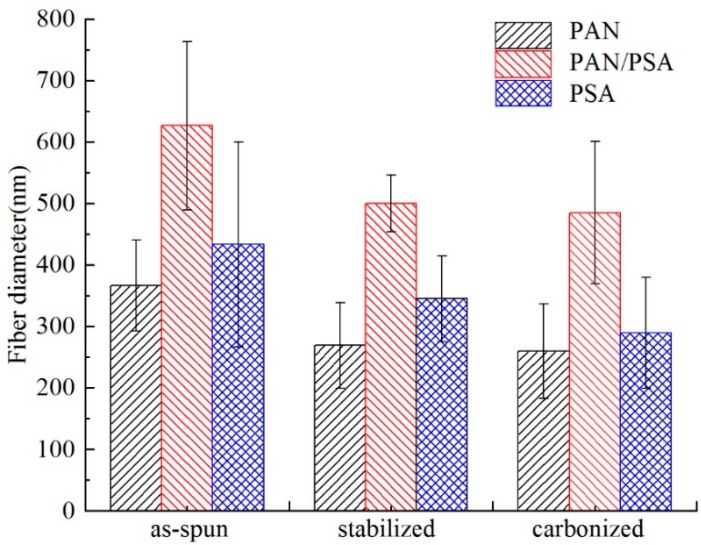
Comparison of the fiber diameter of different materials at different processing stages.

After stabilization, the diameter of the fiber is decreased; and after carbonization, the fiber diameter becomes even smaller. In addition, the diameter is decreased compared with the membrane before carbonization as can be seen in Figure 11 and Figure 12, the fiber diameters from precursors diminished after carbonization, the shrinkage of fiber diameter from as-spun fibers to carbonized fibers for precursor with different materials showed different degrees. As to the three different kinds of membranes, PAN/PSA composite membrane has the largest fiber diameter and PAN has the smallest one at every processing stage.

**Figure 13:**
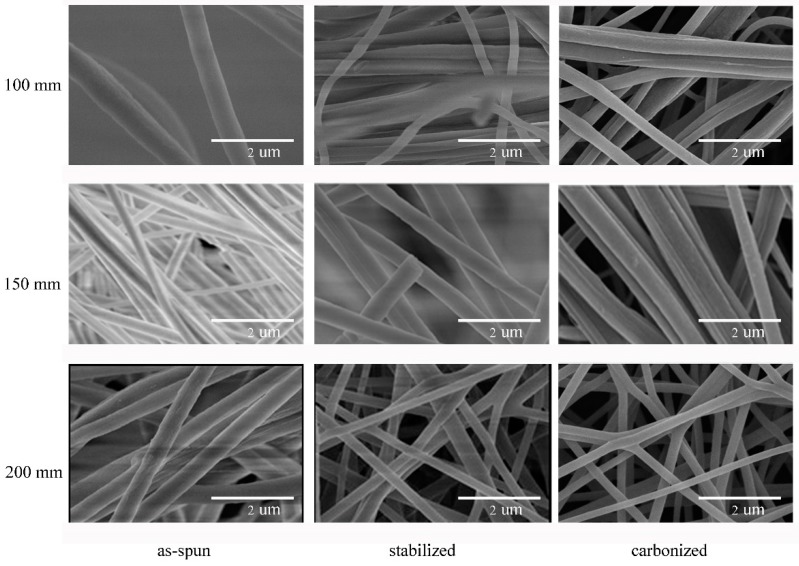
SEM image of membranes of different receiving distance at different processing stages.

**Figure 14:**
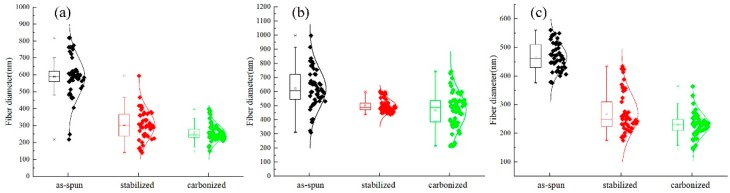
Comparison of the fiber diameter of different receiving distance at different processing stages: (**a**) 100 mm; (**b**) 150 mm; (**c**) 200 mm.

**Figure 15:**
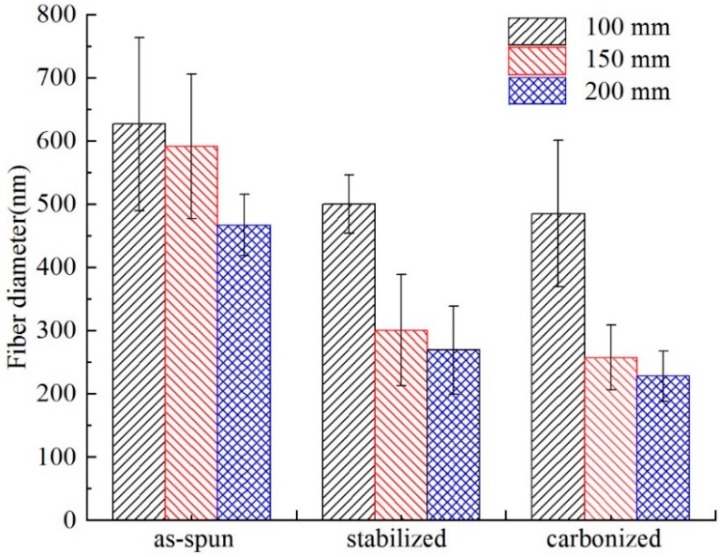
Comparison of the fiber diameter of different receiving distance at different processing stages.

The surface morphology of PAN/PSA nanofibers with the receiving distances before or after stabilized and carbonized stage were illustrated in Figure 13. the statistics and changes of fiber diameter were presented in Figure 14 and Figure 15 respectively, the fiber diameters from precursors diminished after carbonization were decreased significantly. During the spinning process, the increase of the receiving distance makes the electric field draw the polymer jet more completely. When the receiving distance increased from 100 mm to 150 mm and 200 mm, the receiving distance increased by 50% and 100% respectively. It can be ignored that the influence of the receiving distance on the jet speed because when the jet moves in the spinning space, the moving speed is as high as 30 m/s. However, the jet motion in electrospinning has a longer residence time in the electric field, so the nanofibers can be drawn completely, thus the finer fibers were obtained (the as-spun nanofiber diameter decreased from 626 nm to 466 nm).

### 3.4. Thermal Stability

**Figure 16:**
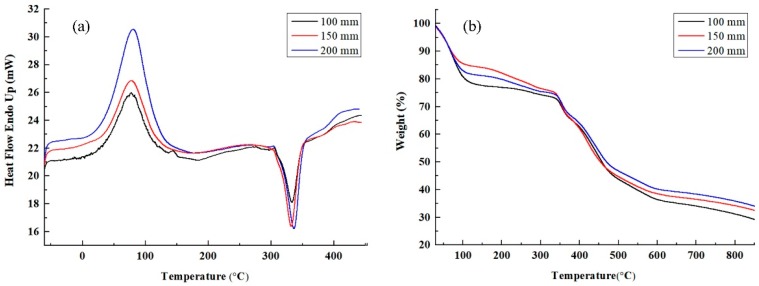
(**a**) DSC and (**b**) TGA curves of fiber membranes with different receiving distances.

As can be seen from the DSC curve in Figure 16a, both the peak width and the peak height at a receiving distance of 200 mm were greater than those with a receiving distance of 150 mm and 100 mm with the same spinning solution, solvent, and voltage for the electrospun PAN/PSA nanofiber membranes. In the case of the same fiber component, the degree of heat transfer and the degree of cyclation are determined by the structure of the fibers. When the receiving distance is 200 mm, the nanofibers were well drawn in the electric field, and the arrangement of fibers inside is relatively compact and has a high orientation so that it has a higher cyclization temperature.

Corresponding to the DSC test results, the same conclusion can also be drawn from the TGA curve of the nanofiber membrane as Figure 16b shows. In the decomposition process of these three fiber membranes, the initial decomposition temperature is basically the same and the decomposition rate did not change much, the difference is the final sample quality residual rate. The final residual mass of the sample increases with increasing receiving distance because the fiber drafting is more complete at 200 mm reception distance, resulting in a tighter fiber structure and improving heat resistance.

### 3.5. Electrical Conductivity

**Figure 17:**
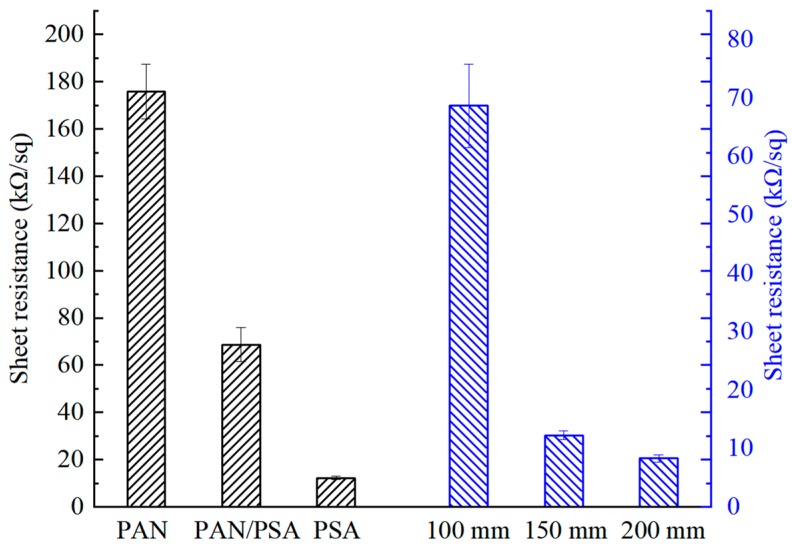
The sheet resistance of different membranes.

As illustrated in Figure 17, the unit of the sheet resistance value for the carbonized fiber membrane is kΩ/sq, the PAN has the largest resistance, the PAN/PSA composite nanofiber membrane is the second, and the PSA has the best conductivity, but the flexibility of PSA carbon fiber membrane was poor in this experiment. As can be seen from the Figure 17, by changing the receiving distance during the preparation process of the PAN/PSA nanofiber membranes, the resistance changed significantly. As the receiving distance increases, the membrane shows a better conductivity. It is considered that, compared with the PAN/PSA fiber membranes with smaller receiving distance, the orientation of the fiber membrane before carbonization is better with the receiving distance of 200 mm. After carbonization, the conductive network between the fibers is relatively distributed evenly, so the resistance is relatively small.

**Figure 18:**
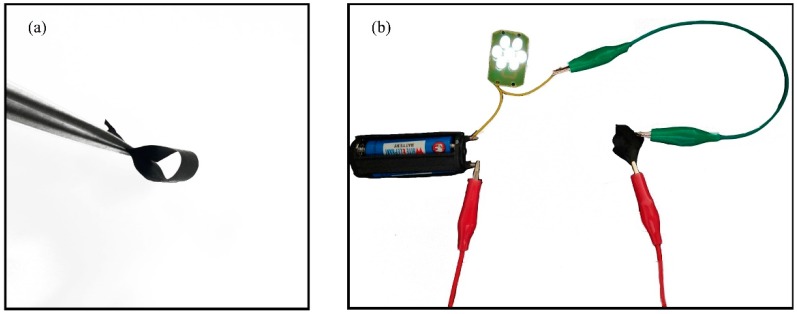
(**a**) Flexibility demonstration of fabricated the PAN/PSA carbonized membrane; (**b**) Performance demonstration using a light-emitting diode wired to PAN/PSA carbonized membrane. The spinning voltage is 20 kV and the receiving distance is 200 mm.

Figure 18 is a representation of the flexibility (**a**) and conductivity (**b**) properties of the membrane. As shown in Figure 18b, the PAN/PSA membrane prepared under a 200 mm receiving distance is connected to a LED light, the LED light has a strong brightness after turning on the power, and the LED light does not light when connecting the uncarbonized membrane, indicating that the conductivity of the PAN/PSA membrane prepared is good in this experiment. It can be seen from the bending test result that the fiber membrane has a good flexibility as shown in Figure 18a.

## 4. Conclusions

In summary, we report a side-by-side electrospinning system to study the effect of spinneret size, spinning voltage, and receiving distance on electric field distribution and intensity by using simulation methods. The results showed that the electric field distribution and intensity can be greatly influenced by the needles thickness, spinning voltage, and receiving distance.

The receiving distance was selected as a research parameter in the experimental verification by using PAN and PSA as the materials, and the prepared membranes were treated under high-temperature for carbonization. It was observed that the membranes spun with different receiving distances show different performance. When the receiving distance was 200 mm, PAN/PSA membrane not only had a good morphology, but also showed good heat resistance, and the electrical conductivity of carbonized membranes was also the best in this study.

## References

[1] Wu T., Zhang J.L., Wang Y.F., Sun B.B., Guo X.R., Morsi Y., Elhamshary H., Elnewehy M., Mo X.M. (2017). Development of dynamic liquid and conjugated electrospun poly(l-lactide-*co*-caprolactone)/collagen nanoyarns for regulating vascular smooth muscle cells growth. J. Biomed. Nanotechnol..

[2] Yu Y., Huang Q.Y., Niu L.Y., Wang D.R., Yan C., She Y.Y., Zheng Z.J. (2017). Waterproof, ultrahigh areal-capacitance, wearable supercapacitor fabrics. Adv. Mater..

[3] Zhang Y., Wang L., Guo Z.Y., Xu Y.F., Wang Y.G., Peng H.S. (2016). High-performance lithium-air battery with a coaxial-fiber architecture. Angew. Chem. Int. Ed..

[4] Zhou G.Y., Xiong T.R., Jiang S.H., Jian S.Y., Zhou Z.P., Hou H.Q. (2016). Flexible titanium carbide–carbon nanofibers with high modulus and high conductivity by electrospinning. Mater. Lett..

[5] Li J.N., Jing P.P., Zhang X.L., Cao D.R., Wei J.W., Pan L.N., Liu Z.L., Wang J.B., Liu Q.F. (2017). Synthesis, characterization and magnetic properties of NiFe_2−x_Ce_x_O_4_ nanoribbons by electrospinning. J. Magn. Magn. Mater..

[6] Wang X.F., Iqbal N., Yu J.Y., Jabeen N., Wazir H.U., Ding B. (2015). In-situ synthesis of carbon nanotubes doped metal-organic frame work for CO_2_ capture. RSC Adv..

[7] He J.X., Lian Y.P., Zhang X.L., Wu Y.C., Liu R.T. (2014). Mass preparation of nanofibers by high pressure air-jet split electrospinning: Effect of electric field. J. Polym. Sci. Part B Polym. Phys..

[8] Vysloužilová L., Buzgo M., Pokorný P., Chvojka J., Míčková A., Rampichová M., Kula J., Pejchar K., Bílek M., Lukáš D. (2017). Needleless coaxial electrospinning: A novel approach to mass production of coaxial nanofibers. Int. J. Pharm..

[9] Kim M.H., Lee W.J., Lee D.H., Ko S.W., Hwang T.I., Kim J.W., Park C.H., Kim C.S. (2018). Development of nanofiber reinforced double layered cabin air filter using novel upward mass production electrospinning set up. J. Nanosci. Nanotechnol..

[10] Huan S.Q., Liu G.X., Han G.P., Cheng W.L., Fu Z.Y., Wu Q.L., Wang Q.W. (2015). Effect of experimental parameters on morphological, mechanical and hydrophobic properties of electrospun polystyrene fibers. Materials.

[11] Lasprilla-Botero J., Álvarez-Láinez M., Lagaron J.M. (2018). The influence of electrospinning parameters and solvent selection on the morphology and diameter of polyimide nanofibers. Mater. Today. Commun..

[12] Casasola R., Thomas N.L., Trybala A., Georgiadou S. (2014). Electrospun poly lactic acid (PLA) fibres: Effect of different solvent systems on fibre morphology and diameter. Polymer.

[13] Anis S.F., Hashaikeh R. (2016). Electrospun zeolite-Y fibers: Fabrication and morphology analysis. Microporous Mesoporous. Mater..

[14] Baqeri M., Abolhasani M.M., Mozdianfard M.R., Guo Q., Oroumei A., Naebe M. (2015). Influence of processing conditions on polymorphic behavior, crystallinity, and morphology of electrospun poly(VInylidene fluoride) nanofibers. J. Appl. Polym. Sci..

[15] Zheng Y.S., Meng N., Xin B.J. (2018). Effects of jet path on electrospun polystyrene fibers. Polymers.

[16] Lai C.C., Lo C.T. (2015). Effect of temperature on morphology and electrochemical capacitive properties of electrospun carbon nanofibers and nickel hydroxide composites. Electrochim. Acta.

[17] Li W.W., Qin X.H. (2014). Effect of relative humidity on the morphology of electrospun gelatin aqueous solutions. Adv. Mater. Res..

[18] Xu H.B., Jiang S.H., Ding C.H., Zhu Y.M., Li J.J., Hou H.Q. (2017). High strength and high breaking load of single electrospun polyimide microfiber from water soluble precursor. Mater. Lett..

[19] Ji D.X., Peng S.J., Lu J., Li L.L., Qin X.H., Yang S.Y., Yang G.R., Srinivasan M., Ramakrishna S. (2017). Design and synthesis of porous channel-rich carbon nanofibers for self-standing oxygen reduction reaction and hydrogen evolution reaction bifunctional catalysts in alkaline medium. J. Mater. Chem. A.

[20] Jin S.X., Xin B.J., Zheng Y.S. (2017). Preparation and characterization of polysulfone amide nanoyarns by the dynamic rotating electrospinning method. Text. Res. J..

[21] Zeng X., Ou Y., Yang Y., Xian C., Xie S.H. (2014). Janus-type thermoelectric Co_3_O_4_/TiO_2_ nanofibers by electrospinning. Nanosci. Nanotechnol. Lett..

[22] Yu D.G., Yang C., Jin M., Williams G.R., Zou H., Wang X., Bligh S.W. (2016). Medicated Janus fibers fabricated using a Teflon-coated side-by-side spinneret. Colloids Surf. B.

[23] Zhu C.Q., Deng W., Pan J.Q., Lu B.A., Zhang J.W., Su Q., Xie E.Q., Lan W. (2013). Structure effect of dual-spinneret on the preparation of electrospun composite nanofibers with side-by-side heterojunctions. J. Mater. Sci. Mater. Electron..

[24] Chen G.Y., Xu Y., Yu D.G., Zhang D.F., Chatterton N.P., White K.N. (2015). Structure-tunable Janus fibers fabricated using spinnerets with varying port angles. Chem. Commun..

[25] Peng L., Jiang S.H., Seuß M., Fery A., Lang G., Scheibel T., Agarwal S. (2016). Two-in-one composite fibers with side-by-side arrangement of silk fibroin and poly(l-lactide) by electrospinning. Macromol. Mater. Eng..

[26] Tong X., Bin-Jie X. (2015). Preparation and characterization of polyester staple yarns nanowrapped with polysulfone amide fibers. Ind. Eng. Chem. Res..

[27] Wu K.J., Yao Y.B., Yu J.C., Chen S.H., Wang X.F., Zhang Y.M., Wang H.P. (2017). Cellulose/aromatic polysulfonamide blended fibers with improved properties. Cellulose.

[28] Zhang X.S., Tang X.N., Wang R., Wang R., Yan X., Shi M.W. (2018). The fire retardant properties and pyrolysis mechanism of polysulfonamide (PSA) fibers. Text. Res. J..

[29] Jin S.X., Xin B.J., Zheng Y.S., Liu S.H. (2018). Effect of electric field on the directly electrospun nanofiber yarns: Simulation and experimental study. Fibers Polym..

[30] Lim B.H., Nirmala R., Navamathavan R., Kim H.Y. (2016). Flexible and conducting carbon nanofibers obtained from electrospun polyacrylonitrile/phosphoric acid nanofibers. J. Nanosci. Nanotechnol..

[31] Xu T., Ding Y.C., Wang Z., Zhao Y., Wu W.D., Hao F., Zhu Z.T. (2017). Three-dimensional and ultralight sponges with tunable conductivity assembled from electrospun nanofibers for a highly sensitive tactile pressure sensor. J. Mater. Chem. C.

[32] Dissanayake M.A.K.L., Divarathne H.K.D.W.M.N.R., Thotawatthage C.A., Dissanayake C.B., Senadeera G.K.R., Bandara B.M.R. (2014). Dye-sensitized solar cells based on electrospun polyacrylonitrile (PAN) nanofibre membrane gel electrolyte. Electrochim. Acta.

[33] Singh G., Rana D., Matsuura T., Ramakrishna S., Narbaitz R.M., Tabe S. (2010). Removal of disinfection byproducts from water by carbonized electrospun nanofibrous membranes. Sep. Purif. Technol..

[34] Liu H., Cao C.Y., Wei F.F., Huang P.P., Sun Y.B., Jiang L., Song W.G. (2014). Flexible macroporous carbon nanofiber film with high oil adsorption capacity. J. Mater. Chem. A.

[35] Efome J.E., Rana D., Matsuura T., Lan C.Q. (2018). Experiment and modeling for flux and permeate concentration of heavy metal ion in adsorptive membrane filtration using a metal-organic framework incorporated nanofibrous membrane. Chem. Eng. J..

[36] Abdalla I., Shen J.L., Yu J.Y., Li Z.L., Ding B. (2018). Co_3_O_4_/carbon composite nanofibrous membrane enabled high-efficiency electromagnetic wave absorption. Sci. Rep..

[37] Zhang L.S., Fan W., Liu T.X. (2016). Flexible hierarchical membranes of WS_2_ nanosheets grown on graphene-wrapped electrospun carbon nanofibers as advanced anodes for highly reversible lithium storage. Nanoscale.

[38] He J.X., Zhao S.Y., Lian Y.P., Zhou M.J., Wang L.D., Ding B., Cui S.Z. (2017). Graphene-doped carbon/Fe_3_O_4_ porous nanofibers with hierarchical band construction as high-performance anodes for lithium-ion batteries. Electrochim. Acta.

[39] Khan W.S., Asmatulu R., Rodriguez V., Ceylan M. (2015). Enhancing thermal and ionic conductivities of electrospun PAN and PMMA nanofibers by graphene nanoflake additions for battery-separator applications. Int. J. Energy. Res..

[40] Mirzaei E., Ai J., Sorouri M., Ghanbari H., Verdi J., Faridi-Majidi R. (2015). Functionalization of pan-based electrospun carbon nanofibers by acid oxidation: Study of structural, electrical and mechanical properties. Fullerenes Nanotubes Carbon. Nanostruct..

[41] Elkhaldi R.M., Guclu S., Koyuncu I. (2016). Enhancement of mechanical and physical properties of electrospun PAN nanofiber membranes using PVDF particles. Desalination Water Treat..

[42] Zhang J.J., Wen H.J., Yue L.P., Chai J.C., Ma J., Hu P., Ding G.L., Wang Q.F., Liu Z.H., Cui G.L. (2017). In situ formation of polysulfonamide supported poly(ethylene glycol) divinyl ether based polymer electrolyte toward monolithic sodium ion batteries. Small..

[43] Ruhland K., Frenzel R., Horny R., Nizamutdinova A., Wüllen L.V., Moosburger-Will J., Horn S. (2017). Investigation of the chemical changes during thermal treatment of polyacrylonitrile and ^15^N-labelled polyacrylonitrile by means of in-situ FTIR and ^15^N NMR spectroscopy. Polym. Degrad. Stab..

[44] Ma Y.N., Zhou T., Su G.H., Li Y., Zhang A.M. (2016). Understanding the crystallization behavior of polyamide 6/polyamide 66 alloys from the perspective of hydrogen bonds: Projection moving-window 2D correlation FTIR spectroscopy and the enthalpy. RSC Adv..

[45] Baruah K., Hazarika S., Borthakur S., Dutta N.N. (2012). Preparation and characterization of polysulfone–cyclodextrin composite nanofiltration membrane: Solvent effect. J. Appl. Polym. Sci..

